# Whether and Why Do We Need a Vaccine Against Atherosclerosis? Can We Expect It Anytime Soon?

**DOI:** 10.1007/s11883-023-01186-z

**Published:** 2024-01-02

**Authors:** Stanisław Surma, Amirhossein Sahebkar, Maciej Banach

**Affiliations:** 1 Department of Internal Medicine and Clinical Pharmacology, Medical University of Silesia, 40-752 Katowice, Poland; 2 Biotechnology Research Center, Pharmaceutical Technology Institute, Mashhad University of Medical Sciences, Mashhad, Iran; 3 Applied Biomedical Research Center, Mashhad University of Medical Sciences, Mashhad, Iran; 4 Department of Biotechnology, School of Pharmacy, Mashhad University of Medical Sciences, Mashhad, Iran; 5 Department of Preventive Cardiology and Lipidology, Medical University of Lodz, 93-338 Lodz, Poland; 6 Cardiovascular Research Centre, University of Zielona Gora, 65-417 Zielona Gora, Poland; 7 Department of Cardiology and Adult Congenital Heart Diseases, Polish Mother’s Memorial Hospital Research Institute (PMMHRI), 93-338 Lodz, Poland

**Keywords:** Atherosclerosis cardiovascular disease, LDL-C, Lipid disorders, PCSK9, Anti-PCSK9 vaccine

## Abstract

**Purpose of Review:**

Atherosclerotic cardiovascular disease (ASCVD) is a leading cause of premature death. Lipid disorders, particularly elevated serum low-density lipoprotein cholesterol (LDL-C), contribute significantly to ASCVD. The risk of developing ASCVD is influenced by the duration of exposure to elevated LDL-C concentrations (cholesterol-years concept). Implementing lipid-lowering treatments based on the principles of “the earlier the better,” “the lower the better,” and “the longer the better” has been shown to reduce cardiovascular risk and significantly extend lifespan. Despite the availability of numerous lipid-lowering drugs, achieving satisfactory control of lipid disorders remains very challenging. Therefore, there is a need for novel approaches to improve treatment adherence.

**Recent Findings:**

One promising solution under investigation is the development of an anti-PCSK9 vaccine, which could be administered annually to provide long-term control over LDL-C concentrations. Experimental studies and the sole clinical trial conducted thus far have demonstrated that the anti-PCSK9 vaccine induces a durable immune response associated with lipid-lowering and anti-atherosclerotic effects. Furthermore, it has exhibited good tolerability and a satisfactory safety profile. However, we still need data from phase 2, 3, and cardiovascular outcome trial to confirm its safety and efficacy and add value in the armamentarium of available and perspective lipid-lowering drugs.

**Summary:**

This article highlights the significance of developing an anti-PCSK9 vaccine and provides an overview of the current knowledge on various anti-PCSK9 vaccines.

## Introduction: Lipid Disorders and ASCVD Risk

**Fig. 1:**
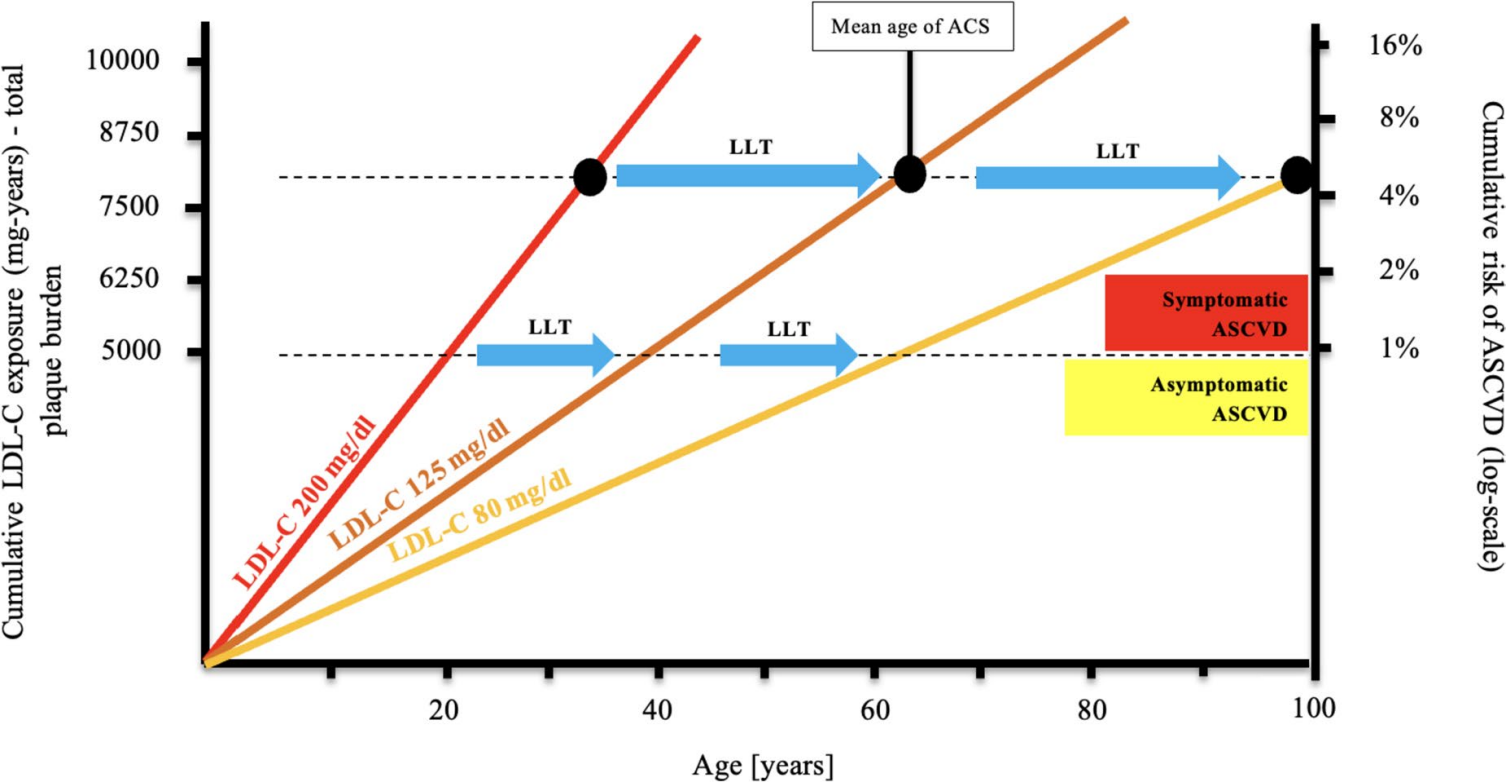
Effect of exposure to different serum LDL-C concentrations on the risk of ASCVD and ACS and the role of lipid-lowering therapy in cardiovascular prevention. Redrawn and modified based on Ference BA et al. J Am Coll Cardiol 2018; 72: 1141-1156 [4]; CC BY-NC-ND license – no permission required. *Abbreviations*: LDL-C, low-density lipoprotein cholesterol; ACS, acute coronary syndrome; ASCVD, atherosclerotic cardiovascular disease; LLT, lipid-lowering therapy

Dyslipidemia is the leading modifiable risk factor and one of the most closely linked markers of atherosclerotic cardiovascular disease (ASCVD), including coronary artery disease (CAD), ischemic stroke, and peripheral arterial disease (PAD) [1]. ASCVD is the leading cause of death worldwide, with even 20 million deaths annually [2]. In 2021, 3.81 million (95% CI, 2.17–5.42) cardiovascular deaths and 3.81 million (95% CI, 2.17–5.42) deaths overall were only attributed to elevated LDL-C serum concentrations, thus might be preventable [1]. It should be emphasized that in the context of ASCVD risk, the duration of lifetime exposure to elevated serum LDL-C concentrations is crucial [3, 4] (Fig. 1).

Therapeutic strategies to reduce low-density lipoprotein cholesterol (LDL-C) serum concentrations are the gold standard in the management of patients with cardiovascular diseases and the most effective cardiac therapy to prevent ASCVD (Fig. 1). The fundamental importance of lipid-lowering treatment in the prevention of ASCVD is indicated by the fact that each decrease in serum LDL-C concentration by 1% is associated with a reduction in cardiovascular risk by about 1%. After 5 years with effective therapy, the risk might be reduced by about 20–25%, and after 40 years, even by 50–55% for each mmol/l of LDL-C [5]. A meta-analysis of 21 studies conducted by Wang et al., covering over 184,000 patients, also showed that the longer the lipid-lowering treatment lasts, the greater the cardiovascular benefits. It was shown that each mmol/l LDL-C serum concentration lowered was associated with a reduced risk of major cardiovascular events of 12% (95% CI, 8–16%) for year 1, 20% (95% CI, 16–24%) for year 3, 23% (95% CI, 18–27%) for year 5, and 29% (95% CI, 14–42%) for year 7 [6]. Everything indicates the need for long-term (and persistent on the part of the patient) reduction of LDL-C serum concentration in patients with lipid disorders, because only then the reduction in cardiovascular risk will increase year by year.

Another fundamental principle of lipid-lowering treatment is the issue of the intensity of lowering LDL-C serum concentration. A meta-analysis of 26 randomized clinical trials conducted by the Cholesterol Treatment Trialists’ (CTT) Collaboration, covering 170,000 patients, showed that more intensive lipid-lowering treatment was associated with an additional reduction in the incidence of major vascular events by 15%, coronary death or non-fatal myocardial infarction by 13%, coronary revascularization by 19%, and ischemic stroke by 16% [7]. The greater benefits of more intensive lipid-lowering treatment are also confirmed by a meta-analysis of 18 randomized clinical trials conducted by Hsu et al., which concluded that more intensive reduction of serum LDL-C concentration was associated with an additional reduction in the risk of cardiovascular events by 24% (RR = 0.76; 95% CI, 0.68–0.85) and the risk of death from any cause by 10% (RR = 0.90; 95% CI, 0.83–0.97) [8]. The duration of exposure to elevated serum LDL-C concentration also plays a key role in the pathogenesis of ASCVD. In a study by Domanski et al., including 4958 participants aged 18–30 years followed for 16 years, it was found that the risk of ASCVD was higher in participants exposed to elevated LDL-C serum concentration at a younger age compared to those at older ages, what emphasizes the importance of optimal LDL cholesterol control from early life [9]. All above confirms that effective (= intensive) lipid-lowering treatment reduces cardiovascular risk and improves the prognosis when carried out in accordance with the principles: “the earlier the better,” “the lower the better,” and “the longer the better” [10, 11].

## Armamentarium of Lipid-Lowering Drugs

The gold standard of lipid-lowering treatment is statins. Other basic LDL-C-lowering drugs include ezetimibe, proprotein convertase subtilisin/kexin 9 (PCSK9) modulators, and bempedoic acid [11]. Currently, it is recommended to use the so-called strong/high intensity statins (pitavastatin, atorvastatin, rosuvastatin) in the maximum tolerated doses, which allows to reduce LDL-C serum concentration by approximately 50% [12, 13]. Depending on the therapeutic goal (determined individually for each patient based on cardiovascular risk and baseline LDL-C concentration), a treatment with appropriate lipid-lowering potency is selected [11]. The use of combinations of lipid-lowering drugs allows to reduce LDL-C serum concentration by even > 85% (strong statin in highest dose + ezetimibe + bempedoic acid + PCSK9 inhibitor) (Table 1) [12]. Based on this in last few years, we replaced recommending high-intensity statin (HIS) therapy with high-intensity lipid-lowering combination therapy or even high-intensity lipid-lowering combination therapy with HIS and ezetimibe, especially after recent reports suggesting that many physicians (even 24%) reduce dose of statin while adding ezetimibe [14].

**Table 1:** How to be effective with lipid-lowering therapy in different patients’ groups at the very high and extremely high CVD risk requiring high-intensity lipid-lowering therapy

Patients with:	Required reduction in serum LDL-C concentration by:	Recommended treatment strategy
ASCVD (e.g., ACS, stroke, and PAD)	50%	HIS
	65%	FDC (HIS + EZE)
	65%	MIS + EZE + BA
	65–70%	HIS + BA
	75%	HIS + FDC (BA + EZE)
	85%	HIS + EZE + PCSK9m
FH	65%	FDC (HIS + EZE)
	65–70%	HIS + BA
	75%	HIS + FDC (BA + EZE)
	85%	HIS + EZE + PCSK9m
	> 85%	HIS + FDC (BA + EZE) + PCSK9m
Complete statin intolerance	40%	FDC (BA + EZE)
	45–65%	PCSK9m
	70%	EZE + PCSK9m
	80–85%	FDC (BA + EZE) + PCSK9i

*Abbreviations*: FDC of high-intensity statin therapy and ezetimibe are a preferable options for all ASCVD patients. *ASCVD*, atherosclerotic cardiovascular disease; *LDL-C*, low-density lipoprotein cholesterol; *ACS*, acute coronary syndrome; *PAD*, peripheral artery disease; *HIS*, high-intensity statin therapy; MIS, moderate-intensity statin therapy; *FDC*, fixed dose combination; *EZE*, ezetimibe; *BA*, bempedoic acid; *PCSK9m*, proprotein convertase subtilisin/kexin 9 modulators/targeted approach therapy (PCSK9 inhibitors + inclisiran)

It is worth emphasizing that a wide range of lipid-lowering drugs also finally allows for individualized therapy, e.g., in patients with statin intolerance and with type 2 diabetes or at a high risk of its occurrence (those with obesity, pre-diabetes). In patients with metabolic disturbances, the most optimal use is pitavastatin (that does not increase new onset diabetes or even has a potential to reduce the risk), ezetimibe, bempedoic acid, and a PCSK9 inhibitor/modulator. The combination of these drugs reduces LDL-C serum concentration by more than 80% and may optimize anti-diabetic treatment [15].

The wide range of lipid-lowering drugs means also that lipid disorders should be hypothetically classified as rare diseases, and the patients over the target should be really infrequent, Unfortunately, this is still not the case.

## Problem with Adhering to Recommendations and Achieving Therapeutic Goals

Despite the availability of excellent lipid-lowering drugs, the possibility of individualization and gradual intensification of treatment and specific treatment guidelines, the control of lipid disorders, and the achievement of therapeutic goals is very insufficient. Only every third patient in Europe, and in Poland and Central and Eastern Europe, every fourth patient achieves the LDL-C goal [16]. The therapeutic target for very high-risk patients (< 55 mg/dl/< 1.4 mmol/l) is met in only 18% of the European population, 17% of the Polish population, and only 13% in Central and Eastern European countries [16, 17]. On the other hand, the set lipid-lowering target in the group of patients with extremely high cardiovascular risk (at the serum concentration of < 40 mg/dl; < 1 mmol/l) is achieved by less than 10% of patients [16, 17]. Trying to figure out the most common of reasons of this ineffectiveness, two things seem to be critically important—applications of moderate-intensity statin therapy in even 50% of patients and almost lack of combination therapy. More recently, the SANTORINI study, which included 9044 patients with high or very high cardiovascular risk from 14 European countries (unfortunately only from the Western Europe), showed only a small improvement with only 20.1% achieved target serum LDL-C concentration in accordance with the current European Society of Cardiology/European Atherosclerosis Society (ESC/EAS) guidelines from 2019 (24% of patients with high risk and 18.6% of patients with very high risk, respectively). Moreover, it was found that 21.8% of patients did not receive any lipid-lowering treatment (23.5% and 21.1%), statin monotherapy was used by 54.3% of patients (58.4% and 52.5%), while combined therapy in 24% patients (18.1% and 26.4%), and most often, it was a combination of statin and ezetimibe [18]. Considering the real prevalence of statin intolerance (which is about 9%, there is no explanations for > 21% of patients at very high CVD risk and those with ASCVD without any lipid-lowering therapy). The CEPHEUS study, covering 33,198 patients from 29 countries across Asia, Western Europe, Eastern Europe, the Middle East, and South Africa, showed that 50.5% of patients reached their target LDL-C serum concentration (62.8% and 33.5% for patients being treated for primary and secondary cardiovascular prevention, respectively). LDL-C goals were achieved in 74.4%, 57.0%, and 25.5% of patients at moderate/moderately high, high, and very high cardiovascular risk, respectively [19]. It is also worth mentioning that the study by Nelson et al., which included 601,934 patients with ASCVD, showed that 49.9% did not use statins, only 22.5% used high intensity statins, and 27.6% used other low to moderate intensity statins [20]. A study by Koenig et al., including real-world data from 865,732 patients using statins, 34,490 using ezetimibe and 1940 using PCSK9i, showed that after 36 months, persistence rates were 20.6% for statins and 22.3% for ezetimibe, and for PCSK9i, 50.9% (much lower than in the Odyssey APPRISE and the SAFEHEART study that presented even > 97% therapy adherence [21, 22]). It was found that in patients with lipid disorders, high non-persistence rates were observed for all lipid-lowering drugs. Persistence rates were the lowest for statins and the highest for PCSK9i [23]. A study by Khachatryan et al., including 73,275 patients with very high cardiovascular risk, showed that higher adherence and/or treatment intensity of lipid-lowering treatment was associated with significantly lower risk of cardiovascular outcomes or all-cause death [24]. In turn, a study by Rodriguez et al., including 347,104 patients with ASCVD, showed that the worse the adherence to lipid-lowering treatment, the higher the risk of death from any cause by 8–30% [25].

**Fig. 2:**
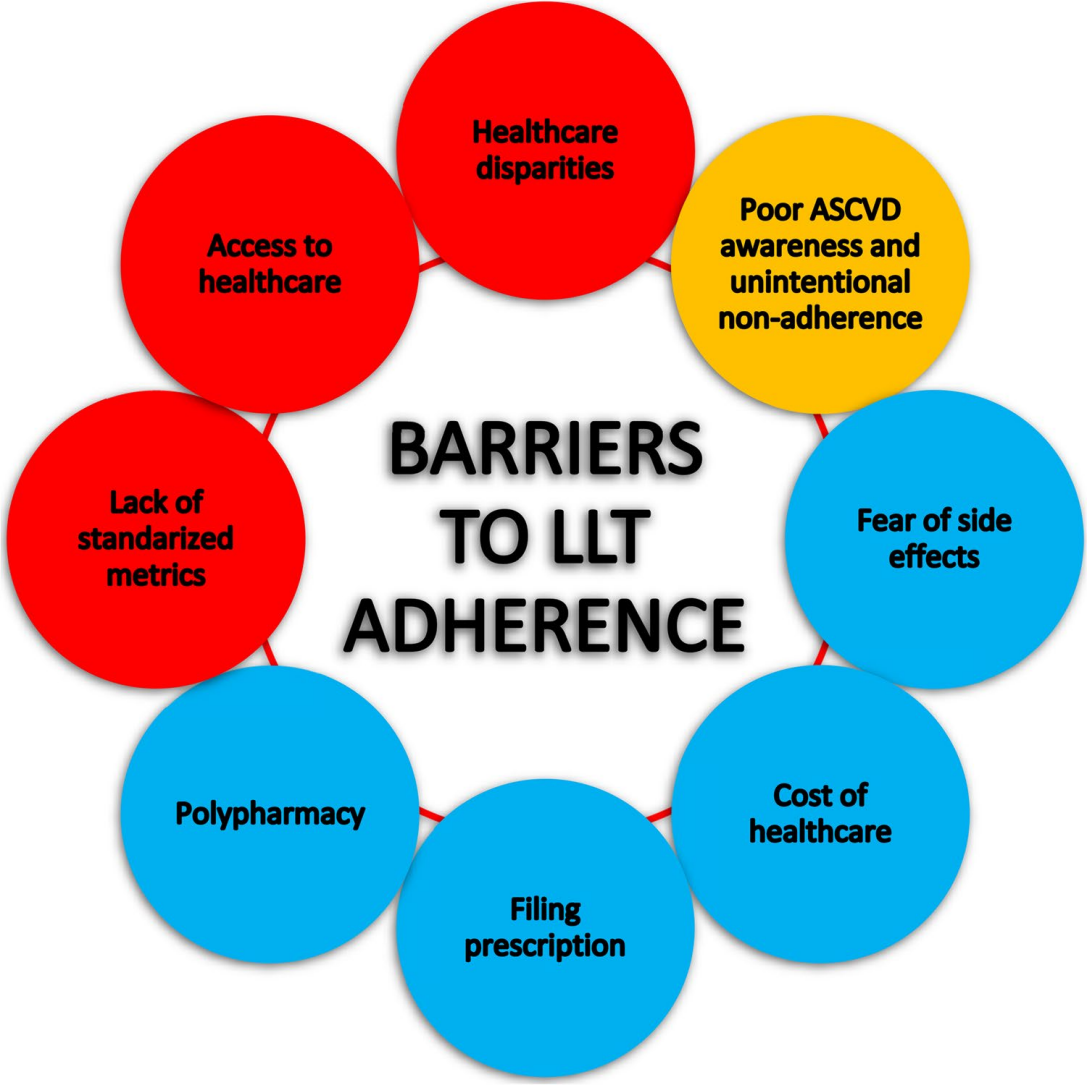
Factors reducing adherence to lipid-lowering treatment. Based on information from [26]. *Abbreviations*: yellow, patient-related factors; red, factors related to healthcare system; blue, factors related to therapy; LLT, lipid-lowering treatment; ASCVD, atherosclerotic cardiovascular disease

Thus, despite the availability of excellent lipid-lowering drugs, many patients with even very high cardiovascular risk do not achieve therapeutic goals. Moreover, both adherence and persistence in the context of lipid-lowering treatment are low, which leads to a worsening of the prognosis of patients. It seems, therefore, that the factors that influence low adherence to lipid-lowering treatment may help to overcome this independent CVD risk factor (Fig. 2) [26].

Particular attention should be paid to issues related to the safety of intensive lipid-lowering treatment and prescription filing (frequency of drug intake). The world’s largest meta-analysis, conducted by Bytyci et al., including 4,143,517 patients, showed that statin intolerance occurred in only 9.1% of them (with only 1–3% for complete statin intolerance), while in an analysis limited to high-quality 112 randomized clinical trials, this percentage was lower and amounted to 4.9% [27, 28]. The same lower ratios were observed when statin intolerance was diagnosed with approved definitions (5.9–7%)—what means than even 93% of patients might be treated with without any safety concerns, what makes statins one of the most tolerable drugs in cardiology. Additionally, a number of factors have been identified that may increase the risk of statin intolerance (e.g., diabetes, liver disease, kidney disease, hypothyroidism, alcohol consumption, obesity, and some drugs) [27]. Moreover, a meta-analysis of 10 randomized clinical trials conducted by Patti et al., including 38,427 patients receiving intensive lipid-lowering therapy (vs. 70,668 patients receiving less intensive treatment), showed that achieving serum LDL-C concentration < 40 mg/dl was not associated with the risk of any adverse events (OR = 1.00; 95% CI: 0.90–1.11), but significantly reduced the risk of cardiovascular events (OR = 0.82; 95% CI, 0.72–0.94) [29]. It is worth emphasizing that the relationship between lowering LDL-C serum concentration and reducing cardiovascular risk does not become a *plateau* at any stage. A further reduction in cardiovascular risk was demonstrated when achieving a serum LDL-C concentration of 8 mg/dl, which was still characterized by a satisfactory safety profile [30]. Intensive lipid-lowering treatment is very safe and is not associated with largely debatable risk of hemorrhagic stroke [31, 32] nor neurocognitive disorders [33] and therefore should be widely used to improve the prognosis of patients [34].

Another issue is the frequency and number of medications used. These factors significantly influence adherence and persistence in treatment, not only lipid-lowering ones. As shown in the previously cited study by Koenig et al., persistence rates were higher for PCSK9i (taken on average 1–2 times/month) compared to statins (taken daily) (50.9% versus 20.6% after 36 months of use) [22]. Long-term statin persistence after 5 years is alarmingly low (< 25%) [35]. The median time to discontinuation of high-intensity statin therapy is 21 months, while that of moderate- or low-intensity statin therapy is 15 months. Subjective side effects are the main reason for discontinuing statin treatment [36, 37]. In the context of the number of medications taken, it is worth mentioning the results of a study by Rea et al., covering 256,012 patients, which showed that compared to those prescribed a two-pill combination lipid-lowering therapy (statin and ezetimibe), those prescribed a single-pill combination (SPC: statin + ezetimibe) had an 87% greater odds of being highly adherent and a 79% lower odds of being poorly adherent to treatment [38]. All this means that there is a strong tendency to develop drugs that are taken less often (Table 2) or to combine in SPCs those that are used every day.

**Table 2:** Evolution of the approach to lipid-lowering treatment

Therapeutic strategy	Mechanism of action	Intervention frequency
Conventional medicines - Statins - Ezetimibe - Bempedoic acid - Fibrates - OMEGA-3 acids - Or combined treatment, preferably in the form of one tablet	Reducing endogenous cholesterol synthesis Reducing cholesterol absorption Other	Daily
Monoclonal antibodies - Alirocumab - Evolocumab - Evinacumab	Reduction in PCSK9 concentration Reduction in ANGPTL3 concentration	1–2 times a month
Antisense oligonucleotides - Volanesorsen - Wupanorsen - Pelacarsen	Reduction of apo(a) or ANGPTL3 or apoCIII production in the liver	Once a week/once a month
Silencing RNA (siRNA) - Inclisiran - Olpasiran	Reduction of apo(a) or PCSK9 production in the liver	Twice a year
Vaccine against PCSK9	Reduction in PCSK9 concentration	Once a year?
Removal of the *PCSK9* gene using the CRISPR-Cas9 method	Reduction of PCSK9 production in the liver	Once in a lifetime?

*Abbreviations*: *PCSK9*, proprotein convertase subtilisin/kexin 9; *ANGPTL3*, angiopoietin-like protein 3; *apoCIII*, apolipoprotein CIII; *apo(a)*, apolipoprotein(a); *CRISPR-Cas9*, clustered regularly interspaced short palindromic repeats

Currently available lipid-lowering drugs that require administration less frequently than daily are PCSK9i (monthly) and inclisiran (2 times/year) [39]. Vaccines against PCSK9 (which will most likely be administered once a year with booster doses every few years even) and genetic modifications involving the removal of the PCSK9 gene (CRISPR, one intervention in a lifetime) appear on the horizon [39].

## PCSK9—Role in Lipoprotein Metabolism and Impact on Cardiovascular Risk

PCSK9 is an important regulatory factor of LDL-C metabolism. Plasma LDL-C are cleared from the plasma mainly through the LDL receptor (LDL-R)-dependent pathway. After LDL-C binds to LDL-R, LDL-C and LDL-R are internalized into clathrin-coated pits and degraded in the lysosome. Secreted PCSK9 binds to epidermal growth factor-like repeat of LDL-R and then increases lysosomal degradation (LDL-C and LDL-R). As a result of the action of PCSK9, the amount of LDL-R on the surface of hepatocytes is reduced, as a result of which LDL-C is not effectively captured from the circulation. When PCSK9 concentration is high, PCSK9 will enhance the degradation of LDL-R in acidic lysosomes and then increased serum LDL concentration. In the absence of PCSK9, LDL-R exists at the cell surface and delivery of LDL-C particles to degradation in acidic endosomes, and then, LDL-R recycled back to the cell surface [40–42].

This means that the higher the concentration of PCSK9, the less LDL-R there is on the surface of hepatocytes and, consequently, the concentration of LDL-C in the serum increases [43]. *PCSK9 loss of function* mutations are associated with low serum LDL-C concentration [44]. A study by Leander et al., including 4,232 participants, showed that increasing serum PCSK9 concentration is associated with future risk of ASCVD even after adjustments for established cardiovascular risk factors [45].

There are currently two strategies available to reduce PCSK9 production in the liver. Alirocumab and evolocumab, which are human monoclonal antibodies (mAbs), capture PCSK9 in the circulation which are then degraded (passive immunization). Inclisiran, a silencing RNA (siRNA), blocks the transcription of the *PCSK9* gene, resulting in a decrease in the amount of PCSK9 produced in the liver. The use of PCSK9 inhibitors allows to reduce the concentration of LDL-C in serum by 50–65%, respectively, and inclisiran by about 50% [3, 11]. PCSK9 inhibitors and inclisiran significantly reduce CVD risk and improve patient prognosis [46, 47]. In the next few years, probably, also the oral form of PCSK9 inhibitors will be available [48].

Thus, therapeutic intervention by reducing PCSK9 activity is very effective. However, these drugs are expensive and not always available—in most of the countries within less or more restricted reimbursement programs. Moreover, long-term clinical use of mAbs has limitations such as relatively short in vivo half-life, requiring frequent administration and high cost, some tolerability issues, and possible induction of host anti-mAb. Hence, the development of a vaccine against PCSK9 (active immunization) that is cheap and widely available and requires administration once a year (with subsequent boosters) is an important direction of current research in lipidology.

## Anti-PCSK9 Vaccine—Current State of Knowledge

Various types of vaccines against PCSK9 are currently being tested: peptide vaccines (epitope vaccines), nanoliposome vaccines containing the PCSK9 epitope conjugated to the Tetanus epitope, virus-like particle (VLP)-peptide vaccines anti-PCSK9, etc. The main differences include the carrier used (nanoliposomes, VLPs), the presence of an adjuvant (tetanus), valenty, and the length of the antigen PCSK9 [49].

### Peptide Vaccine Against PCSK9

The study by Galabova et al. assessed the effectiveness and safety of peptide-based anti-PCSK9 vaccines using wild-type mice, LDLR^+/−^ mice and rats [50]. It was shown that the vaccine stimulated the production of specific anti-PCSK9 antibodies, which led to a reduction in total cholesterol and LDL-C serum concentration by 30% and 50%, respectively. The humoral immune response in mice lasted for a year and was accompanied by a lipid-lowering effect. In all animals tested, administration of the vaccine was well tolerated and safe [50]. A peptide vaccine against PCSK9 was also the subject of a study by Ataei et al. In the study by these authors, the immunogenic potential of a vaccine consisting of the PCSK9 peptide and 0.4% alum adjuvant was assessed in albino mice. Under the influence of the intervention, IgG antibodies against PCSK9 were produced, which resulted in a decrease in the concentration of PCSK9 in the serum by 21–22% and a reduction in the binding of PCSK9 to LDL-R by 26–34% [51]. The lipid-lowering effectiveness of a peptide vaccine anti-PCSK9 conjugated with keyhole limpet hemocyanin (KLH) as a carrier protein in mice was also demonstrated by Kawakami et al. [52]. A study by Landlinger et al. showed that the use of the AT04A peptide vaccine against PCSK9 in APOE*3Leiden.CETP mice fed a western-type diet for 18 weeks was associated with the formation of anti-PCSK9 antibodies, a decrease in PCSK9 concentration (by 57% in 4 week and 24% at week 18), a 53% reduction in total cholesterol, and a reduction in LDL-C serum concentrations [53]. Moreover, the AT04A vaccine showed a reduction in the production of various pro-inflammatory factors, such as serum amyloid A (SAA), macrophage inflammatory protein-1b (MIP-1b/CCL4), macrophage-derived chemokine (MDC/CCL22), cytokine stem cell factor (SCF), and vascular endothelial growth factor A (VEGF-A) [53]. The AT04A vaccine slowed down the progression of atherosclerosis, showing a decrease in atherosclerotic lesion area (− 64%) in aorta and aortic inflammation as well as in more lesion-free aortic segments (+ 119%), compared with control [53]. The favorable results of this experiment were the basis for conducting the study in a clinical setting. Currently, the only vaccine tested in a clinical trial is AT04A and AT06A, which are two AFFITOPE^®^ peptide vaccines (NCT02508896). In a randomized, placebo-controlled phase I clinical trial by Zeitlinger et al., 72 healthy volunteers with a mean fasting LDL-C serum concentration at baseline of 117.1 mg/dl underwent an intervention consisting of three priming immunizations at weeks 0, 4, and 8 to receive a single booster immunization at week 60 of either AT04A, AT06A, or placebo [54••]. It was shown that the AT04A and AT06A vaccines had a good tolerability profile. The most frequently reported adverse events (AEs) were fatigue, headache, and myalgia in 75% of participants in the AT06A group and 58% and 46% of participants in the placebo and AT04A groups, respectively. The most frequently reported acute adverse effects (TEAEs) were injection site reactions (63%), most often of which they were transient and mild to moderate in nature. Both vaccines induced a strong and long-lasting immune response against PCSK9. Nevertheless, only AT04A had a lipid-lowering effect. It was found that during the whole study period, the mean LDL-C concentration reduction for the AT04A group versus placebo was only − 7.2% (95% CI, − 10.4 to − 3.9, *p* < 0.0001) [54••].

### Nanoliposome Anti-PCSK9 Vaccine

**Fig. 3:**
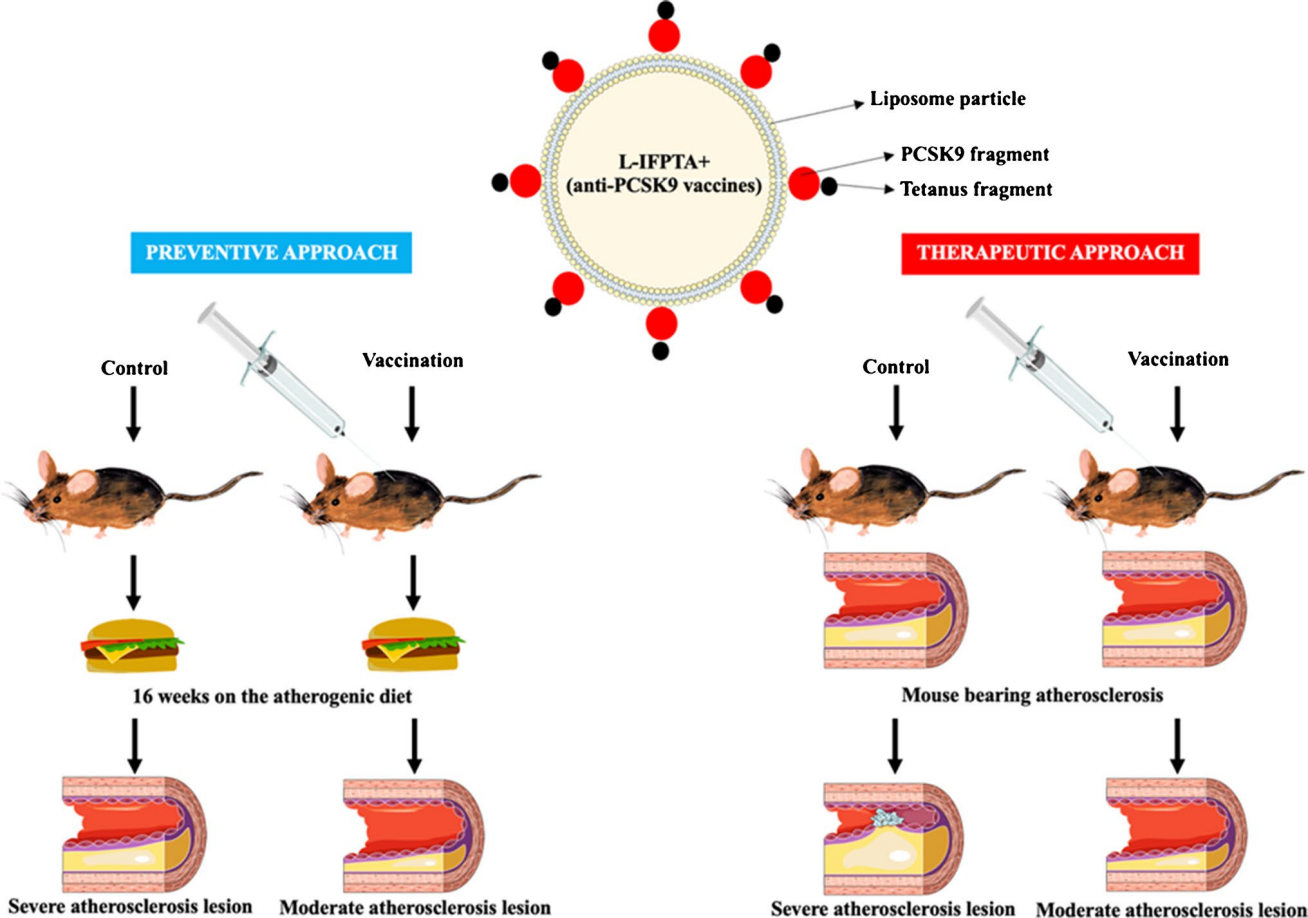
Preventive and therapeutic approach to the use of L-IFPTA+ vaccine against PCSK9. Based on research results [55–60]. *Abbreviations*: PCSK9, proprotein convertase subtilisin/kexin 9

The study by Momtazi-Borojeni et al. assessed the effectiveness of nanoliposome anti-PCSK9 vaccine in C57BL/6 mice with hypercholesterolemia. A nanoliposomal vaccine was used containing peptide construct termed immunogenic fused PCSK9-tetanus (IFPT) that was displayed on the surface of liposome nanoparticles (L-IFPT) and mixed into alum adjuvant (L-IFPTA+). Different vaccine formulations (IFPT, L-IFPT, L-IFPTA+, and empty nanoliposomes) were administered subcutaneously 4 times at 2-week intervals. The L-IFPTA+ vaccine was shown to induce the most pronounced IgG response against PCSK9. Plasma concentration of PCSK9 in vaccines and control groups were 61.37 ± 5.53 ng/ml and 101.5 ± 8.04 ng/ml, respectively [55, 56•]. Significant reductions in PCSK9 binding to LDL-R were found. The vaccination led to an increase in the number of LDL-R on the surface of hepatocytes. The L-IFPTA+ vaccine had the greatest lipid-lowering effect. This vaccine reduced total cholesterol concentration by − 82.5 ± 7.3% (*p* = 0.002) and LDL-C concentration by − 88.14 ± 5.6%. Moreover, mice immunized with the L-IFPTA+ vaccine exhibited a significantly decreased atherosclerotic lesion size (24.25%, *p* = 0.002) in the aortic arch and intima-to-media thickness (30.2%, *p* = 0.007), compared with the negative controls after week 16 using an atherogenic diet. The anti-inflammatory properties of the L-IFPTA+ vaccine were also found (Th2 cells and IL-4 cytokine were significantly increased in splenocytes of vaccinated mice). The vaccine was characterized by a satisfactory safety profile [56•]. The primary challenge in designing a secure vaccine against self-antigens like PCSK9 is effectively overcoming B-cell tolerance without triggering the destructive autoreactive T-cell response [55]. Therefore, it is crucial in vaccine development to exclude peptide antigens that might elicit specific T-cell responses. However, B cells require assistance from CD4+ T helper (Th) cells for efficient activation and differentiation into enduring plasma and memory cells. One strategy to induce sufficient autoantibody generation is to physically link a B-cell epitope of a self-antigen to a foreign Th epitope [55–60]. To achieve this, the L-IFPTA vaccine incorporates two distinct epitopes from PCSK9 and tetanus toxin proteins. The PCSK9 fragment serves as a B-cell epitope, mimicking an N-terminal sequence responsible for PCSK9 binding to LDLR. The amino acid sequence of the PCSK9 fragment was designed using AFFITOPE technology in a way different from the native sequence, making it identifiable as foreign by the immune system and thus overcoming self-tolerance [55, 56•]. The PCSK9 fragment exhibits a close similarity between humans and rodents, enabling vaccine-generated antibodies to potentially block the PCSK9/LDLR interaction in both species. To enhance the CD4+ T-cell response, a tetanus peptide as a foreign Th epitope was coupled with the PCSK9 fragment. The L-IFPTA vaccine adsorbed to Alum adjuvant can inhibit PCSK9-specific T-cell activation while promoting a tetanus-specific T-cell response that enhances PCSK9-specific B-cell activation without safety concerns [55, 56•]. The series of studies with L-IFPTA+ vaccine indicates its potential important role in the atherosclerosis prevention in different models [56•, 57–60] (Fig. 3).

It is worth emphasizing that the L-IFPTA+ vaccine elicited higher and more durable titers of anti-PCSK9 antibody compared with the peptide vaccine [55, 56•]. In one of the studies, the investigators assessed the effectiveness and safety of the L-IFPTA+ vaccine administered four times with bi-weekly intervals in C57BL/6 mice on the background of a severe atherogenic diet and poloxamer 407 (thrice weekly) injection [55, 56•, 57]. It was shown that L-IFPTA+ stimulated the production of antibodies against PCSK9, which resulted in a 58.5% reduction in plasma PCSK9 concentration. The binding of PCSK9 to LDL-R was also significantly reduced. After 8 weeks, L-IFPTA+ was shown to reduce total cholesterol, LDL-C, and VLDL-C by up to 44.7%, 51.7%, and 19.2%, respectively [55]. Over 16 weeks post-prime immunization, the reduction in LDL-C concentration was still significant and reached 42% [55]. Importantly, the use of L-IFPTA+ was associated with a significantly decrease in the atherosclerotic lesion size (39.13%, *p* = 0.016) and IMT (46%, *p* = 0.003) in aortic arc compared with the control. In splenocytes, under the influence of L-IFPTA+, there was an increased number of IL-10-producing cells and fewer IFN-γ-producing cells [55]. This indicates a potential important role of the anti-PCSK9 L-IFPTA+ vaccine in the treatment of existing atherosclerosis (Fig. 3). A study by Ataei et al. showed that the use of L-IFPTA+ vaccine in mice led to a reduction in the level of miR-27a. miR-27a has been shown to decrease LDL-R levels by directly binding to its 3′-untranslated region (UTR) and indirectly by enhancing PCSK9 [57].

In the next stage, Momtazi-Borojeni et al. assessed the effectiveness and safety of the L-IFPTA+ vaccine in a preclinical study involving non-human primates—five male rhesus macaque monkeys. The animals were vaccinated with L-IFPTA+ four times with bi-weekly intervals. It was demonstrated that L-IFPTA+ induced an effective and safe immune response [58]. A reduction in the degree of PCSK9 binding to LDL-R by − 33 ± 7% was observed. There was no significant effect of L-IFPTA+ on liver and kidney function parameters as well as inflammatory parameters. Some lipid-lowering effects were demonstrated (reductions in plasma concentrations of total cholesterol, LDL-C, VLDL-C and triglycerides by 11.6 ± 36%, 16 ± 28%, 22 ± 53%, and 24 ± 51%, respectively, while HDL-C was slightly increased by 2 ± 64%); however, it was not significant [58]. The modest lipid-lowering effect of the L-IFPTA+ vaccine in this study on serum LDL-C (16% reduction) and PCSK9 (33% inhibition) is not surprising because it was found that differences in circulating PCSK9 concentration could only explain 7–8% of the variation circulating LDL-C concentration. Moreover, the normolipidemic status of the animals and the small sample size could also have contributed to the limited lipid-lowering effect [58]. Another study by Momtazi-Borojeni et al. assessed the effect of IFPT vaccine against PCSK9 on carbohydrate and lipid metabolism in diabetic rats. Healthy rats received four doses of IFPT at 2-week intervals. The rats were then administered streptozotocin intraperitoneally to induce diabetes. The vaccine led to the development of a strong immune response against PCSK9, which was associated with a reduction in PCSK9 concentration by 58% and LDL-C in plasma by nearly 27%. The binding of PSCK9 to LDL-R was also reduced by 30% [59]. In the context of the impact of IFPT on carbohydrate metabolism, it was found that vaccinated rats were characterized by a 49% lower fasting glucose concentration (FGB), improved glucose tolerance in the oral glucose tolerance test (OGTT), and a greater hypoglycemic response in the insulin tolerance test (ITT) (reduced glucose concentration by 49% compared to rats not vaccinated with IFPT). Therefore, anti-PCSK9 vaccination with IFPT vaccine may have antidiabetic effects and improve the lipid profile in diabetes [59]. A vaccine composed of a peptide construct containing PCSK9 and tetanus epitopes was also tested for toxicity by Momtazi-Borojeni et al. in a study using healthy male and female albino mice. Vaccination was planned based on 4 subcutaneous injections of the vaccine formulation (10 μg/mouse) in bi-weekly intervals. There was no effect of this vaccine on the lipid profile, renal function, liver function, or hematological disorders. Moreover, in histopathological examinations of various tissues including the heart, liver, kidney, spleen, and brain, no significant differences were found between vaccinated and control mice. Thus, a satisfactory safety profile of the peptide vaccine containing PCSK9 and tetanus epitopes was found [60].

In the recent study, presented at the European Society of Cardiology Congress 2023, the investigators aimed to find whether the PCSK9 inhibition using the antiPCSK9 vaccine can affect the hs-CRP level and the oxidative stress during systemic inflammation. The serum concentration of hs-CRP in the vaccine, CFA (mice treated with Freund’s complete adjuvant to induce inflammation), and sham (non-treated mice) groups were 14.65 ± 4.66 mg/l, 17.84 ± 5.37 mg/l, and 6.5 ± 2.02 mg/l, respectively. To increase hs-CRP, all mice were subjected to the CFA challenge after the vaccination plan. The statistical analysis indicated that the level of hs-CRP was significantly increased in the vaccine and CFA groups by 225% (*p* = 0.037) and 274% (*p* = 0.004), respectively when compared with the sham group; however, it was non-significantly decreased in the vaccine group in comparison with the CFA group (by 18%, *p* = 0.520). The pro-oxidant antioxidant balance (PAB; oxidative stress) values in the vaccine, CFA, and sham groups were 54.22 ± 10.93 HK, 53.19 ± 9.8 HK, and 30.8 ± 6.7 HK, respectively. The PAB value was significantly increased in the CFA group (by 72.7%, *p* < 0.001) and the vaccine group (by 76%, *p* < 0.001) when compared with the sham group with no significant difference between the vaccine and CFA groups. These results indicate that the antiPCSK9 vaccine, despite its significant efficacy in inhibiting PCSK9 function, could not protect against CFA-induced acute inflammation and oxidative stress in mice [61].

### Virus-Like Particle (VLP)-Peptide Vaccines Anti PCSK9

A different approach was used by Pan et al., using virus-like particle (VLP)-peptide vaccines anti-PCSK9 in both Balb/c and LDLR^+/−^ mice [62]. The PCSK9Qβ-003 vaccine (Qβ bacteriophage VLP-peptide vaccine) caused the production of high titers of IgG antibodies against PCSK9, reduced the concentration of PCSK9 in plasma, and increased the expression of LDL-R in the liver. Additionally, PCSK9Qβ-003 vaccine was found to decreased plasma total cholesterol in both Balb/c mice and LDLR^+/−^ mice. Additionally, PCSK9Qβ-003 vaccine injection was associated with significant up-regulation of sterol-regulatory element-binding protein-2 (SREBP-2), hepatocyte nuclear factor 1α (HNF-1α), and 3-hydroxy-3-methylglutaryl coenzyme A (HMG-CoA) reductase in LDLR^+/−^ mice. No obvious immune injury was detected in animal disease [62]. The study by Wu et al. showed that the use of the PCSK9Qβ-003 vaccine was characterized by antifibrotic effects related to the regulation of fatty acid β-oxidation in mice with renal fibrosis and hypercholesterolemia [63]. A very interesting study by Fowler et al. assessed the effectiveness and safety of VLP-based vaccine against PCSK9 in mice and non-human primates [64]. The vaccine used was monovalent or bivalent targeting two distinct epitopes on PCSK9. It has been shown that in both mice and macaques, a bivalent VLP vaccine targeting two distinct epitopes on PCSK9 elicited strong and durable antibody responses and lowered cholesterol concentration. Importantly, in macaques, a VLP vaccine targeting a single PCSK9 epitope was only effective at lowering LDL-C concentration in combination with statins, whereas immunization with the bivalent vaccine lowered LDL-C without requiring statin co-administration. The intervention used was safe [64]. In addition to the vaccine valency, the length of the PCSK9 antigens may also be important. The study by Goksøyr et al. compared the effectiveness of a VLP vaccine containing shorter versus full-length (FL) PCSK9 antigens in an experimental model using BALB/c mice and murine Hepa1-6 hepatocytes [65]. It was shown that the anti-PCSK9 FL VLP vaccine, compared to those containing shorter peptide antigens, induces greater clearance of PCSK9 from plasma, which is related to greater opsonization of PCSK9 by IgG and not the formation of immune complexes (as is the case with the use of anti-PCSK9 mAb and vaccines containing shorter PCSK9 antigen). It was also observed that the FL VLP anti-PCSK9 vaccine reduced the concentration of total cholesterol and triglycerides to a greater extent in the tested mice [65]. The study by Crossey et al. used (VLP)-peptide vaccines against PCSK9 in Balb/c mice and rhesus macaques. The vaccine led to the production of IgG antibodies against PCSK9, which resulted in a significant reduction in the concentration of total cholesterol, free cholesterol, phospholipids, and triglycerides [66]. Finally, the study by Ortega-Rivera et al. compared, among others, the lipid-lowering effectiveness of VLP vaccines against apoB, CETP, and PCSK9 in mice. It was shown that the anti-PCSK9 VLP vaccine had the most pronounced lipid-lowering effect [67].

It is worth mentioning that the vaccine against atherosclerosis may be directed not only against PCSK9. It was shown that vaccine against heat shock protein 25 (HSP25; in humans the ortholog of HSP27 is HSP25) in ApoE^−/−^ mice led to an increase in LDL-R expression, a decrease in PCSK9 concentration in the blood, and a decrease in inflammation [68]. In Table 3, the current status of PCSK9 vaccine studies was summarized.

**Table 3:** Studies on a vaccine against PCSK9—current status (Nov 2023)

Vaccine	Author; year; ref	Evaluated parameters	Research stage
Peptide vaccine against PCSK9	Galabova et al., 2014 [50]	- Immunogenicity - Lipid-lowering effect - Metabolic effect - Tolerance and safety - Anti-atherosclerotic effect	- Experimental: mice and rats
	Ataei et al., 2023 [51]		- Experimental: mice
	Kawakami et al., 2018 [52]		- Experimental: mice
	Landlinger et al., 2017 [53]		- Experimental: mice
	Zeitlinger et al., 2021 [54]	- Immunogenicity - Lipid-lowering effect - Tolerance and safety	- Randomized, placebo controlled, clinical trial: phase I
Nanoliposome anti-PCSK9 vaccine	Momtazi-Borojeni et al., 2021, 2023 [55, 56•, 58–61]	- Immunogenicity - Lipid-lowering effect - Metabolic effect - Preventive effect - Tolerance and safety - Anti-atherosclerotic effect	- Experimental: mice; macaque monkeys; rats - A (pre)clinical trial phase I involving healthy volunteers is currently ongoing
Virus-like particle (VLP)-peptide vaccines anti PCSK9	Pan et al., 2017 [62]	- Immunogenicity - Lipid-lowering effect - Metabolic effect - Tolerance and safety	- Experimental: mice
	Wu et al., 2020 [63]		- Experimental: mice
	Fowler et al., 2023 [64]		- Experimental: mice; macaque monkeys
	Goksøyr et al., 2022 [65]		- Experimental: mice; hepatocyte
	Crossey et al., 2015 [66]		- Experimental: mice; macaque monkeys
	Ortega-Rivera et al., 2021 [67]		- Experimental: mice

*PCSK9*, proprotein convertase subtilisin/kexin 9

## Conclusions and Future Perspective

In conclusion, the development of vaccines targeting PCSK9 shows promising results in reducing cholesterol concentrations and preventing atherosclerosis. Peptide vaccines and nanoliposome vaccines have been investigated, demonstrating their ability to stimulate the production of specific anti-PCSK9 antibodies. Peptide-based vaccines have shown lipid-lowering effects and a reduction in PCSK9 concentration, leading to decreased cholesterol and LDL-C concentrations. Nanoliposome vaccine, the L-IFPTA+ vaccine, has exhibited strong immunogenic responses and significant reductions in PCSK9 binding to LDL-R. These vaccines have also shown significant lipid-lowering effects, reducing total cholesterol and LDL-C serum concentrations. Furthermore, the L-IFPTA+ vaccine has demonstrated a decrease in atherosclerotic lesion size and intima-to-media thickness, along with slight anti-inflammatory properties. Clinical trials involving peptide vaccines, such as AT04A and AT06A, have shown good tolerability and a lipid-lowering effect. The L-IFPTA+ vaccine has exhibited a satisfactory safety profile in preclinical studies involving mice and non-human primates. VLP-peptide vaccines targeting PCSK9 have also shown promising results in reducing cholesterol serum concentration. Studies have demonstrated the production of anti-PCSK9 antibodies, decreased PCSK9 concentration, increased LDL-R expression, and significant reductions in total cholesterol. Bivalent VLP vaccines targeting multiple PCSK9 epitopes have exhibited strong and durable antibody responses, effectively lowering cholesterol serum concentration. The use of full-length PCSK9 antigens in VLP vaccines has shown enhanced clearance of PCSK9 and greater reductions in cholesterol and triglyceride concentrations.

The anti-PCSK9 vaccine offers not only lipid-lowering effects but also the potential to improve treatment adherence. Long-term commitment and poor compliance associated with current therapies [21, 69, 70] can be addressed by the convenience of an annual vaccine administration, although, in early stages, the available evidence suggests the vaccine’s value in lipid-lowering. However, there is still a lot to do, and further human research and trials are needed to confirm and establish its short (after first dosing) and long-term efficacy, safety, optimal dosing, cost-effectiveness, and feasibility of large-scale production. Thus, the anti-PCSK9 vaccine holds promise in achieving sustained LDL-C control and reducing the burden of ASCVD. Continued research is necessary to determine its role in preventing and managing cardiovascular disease. If the studies are successful, in just a several years, a vaccine against atherosclerosis may completely effectively change the prevention and management of ASCVD, the biggest killer in the world [12, 71–74].

## Data Availability

No datasets were generated or analyzed during the current study.
