# Correction to: Whether and Why Do We Need a Vaccine Against Atherosclerosis? Can We Expect It Anytime Soon?

**DOI:** 10.1007/s11883-024-01189-4

**Published:** 2024-01-22

**Authors:** Stanisław Surma, Amirhossein Sahebkar, Maciej Banach

**Affiliations:** 1 Department of Internal Medicine and Clinical Pharmacology, Medical University of Silesia, 40-752 Katowice, Poland; 2 Biotechnology Research Center, Pharmaceutical Technology Institute, Mashhad University of Medical Sciences, Mashhad, Iran; 3 Applied Biomedical Research Center, Mashhad University of Medical Sciences, Mashhad, Iran; 4 Department of Biotechnology, School of Pharmacy, Mashhad University of Medical Sciences, Mashhad, Iran; 5 Department of Preventive Cardiology and Lipidology, Medical University of Lodz, 93-338 Lodz, Poland; 6 Cardiovascular Research Centre, University of Zielona Gora, 65-417 Zielona Gora, Poland; 7 Department of Cardiology and Adult Congenital Heart Diseases, Polish Mother’s Memorial Hospital Research Institute (PMMHRI), 93-338 Lodz, Poland

**Correction to: Current Atherosclerosis Reports**

10.1007/s11883-023-01186-z

The original version of the article contained a typo in Figure3 (mistaken reversal of the place of images). On the left side of Figure 3, in the "preventive approach," atherosclerotic lesions in the control group should be "severe atherosclerosis lesion," while in the vaccinated group it should be "moderate atherosclerosis lesion." The image of the cross-section of the vessel affected by atherosclerosis has been corrected and the explanations have been improved.

The original version has been corrected.

